# The Novel DNA Binding Mechanism of Ridinilazole, a Precision *Clostridiodes difficile* Antibiotic

**DOI:** 10.1128/aac.01563-22

**Published:** 2023-04-24

**Authors:** Clive S. Mason, Tim Avis, Chenlin Hu, Nabeetha Nagalingam, Manikhandan Mudaliar, Chris Coward, Khurshida Begum, Kathleen Gajewski, M. Jahangir Alam, Eugenie Bassères, Stephen Moss, Stefanie Reich, Esther Duperchy, Keith R. Fox, Kevin W. Garey, David J. Powell

**Affiliations:** a Summit Therapeutics, Cambridge, United Kingdom; b Department of Pharmacy Practice and Translational Research, University of Houston College of Pharmacy, Houston, Texas, USA; c Department of Biology & Biochemistry, University of Houston Colleges of Natural Sciences and Mathematics, Houston, Texas, USA; d Domainex Ltd., Cambridge, United Kingdom; e Summit Therapeutics, Abingdon, United Kingdom; f School of Biological Sciences, University of Southampton, Southampton, United Kingdom

**Keywords:** *Clostridium difficile*, antibiotic, mechanisms of action

## Abstract

Clostridioides difficile infection (CDI) causes substantial morbidity and mortality worldwide with limited antibiotic treatment options. Ridinilazole is a precision bisbenzimidazole antibiotic being developed to treat CDI and reduce unacceptably high rates of infection recurrence in patients. Although in late clinical development, the precise mechanism of action by which ridinilazole elicits its bactericidal activity has remained elusive. Here, we present conclusive biochemical and structural data to demonstrate that ridinilazole has a primary DNA binding mechanism, with a co-complex structure confirming binding to the DNA minor groove. Additional RNA-seq data indicated early pleiotropic changes to transcription, with broad effects on multiple C. difficile compartments and significant effects on energy generation pathways particularly. DNA binding and genomic localization was confirmed through confocal microscopy utilizing the intrinsic fluorescence of ridinilazole upon DNA binding. As such, ridinilazole has the potential to be the first antibiotic approved with a DNA minor groove binding mechanism of action.

## INTRODUCTION

Clostridioides difficile infection (CDI) is a major cause of morbidity and mortality, with nearly 13,000 deaths in the United States alone in 2017 (1). Antibiotic treatment options are limited and result in unacceptably high CDI recurrence rates of up to 30% (2, 3) and with a higher likelihood of death at each recurrence (4–6). Current standard of care antibiotics belong to mechanistic classes that generally possess broad spectra of activity. While these antibiotics can effectively kill vegetative C. difficile cells, the further broad collateral damage to the gut microbiota that they induce creates conditions permissive to further spore germination, CDI disease recurrence, and potential colonization with other human pathogens, including multidrug-resistant organisms (7).

Ridinilazole (RDZ), currently in clinical development, has been developed specifically to treat CDI and address CDI-associated recurrence through a targeted, precision approach. It has potent *in vitro* activity against C. difficile clinical isolates (8, 9), but minimal disruption of other gut bacteria and their metabolites that contribute to CDI colonization resistance (10, 11). Ridinilazole also preserves secondary bile acids which play a key role in suppressing the growth of toxin producing vegetative C. difficile cells (12).

The mechanism of action through which ridinilazole works has yet to be fully elucidated. Unlike most currently used antibiotics which are natural-product based, ridinilazole is a symmetrical small molecule drug with a head-to-head bisbenzimidazole core. This structure is similar to nonsymmetrical, head-to-tail bisbenzimidazole Hoechst dyes, and ridinilazole has similar DNA-binding and intrinsic fluorescence properties (13).

In this work, we have utilized these fluorescence properties to study the binding of ridinilazole to one prospective mechanistic target, C. difficile genomic DNA. Biochemical, structural, and cellular imaging studies indicate a primary DNA binding mechanism. Transcriptomics studies have provided a rationale for antibacterial activity of ridinilazole on C. difficile vegetative cells. In combination, we present a conclusive array of data to indicate that ridinilazole exerts its antibiotic effect through a primary DNA binding mechanism which results in transcriptional dysregulation and C. difficile cell death.

## RESULTS

### Fluorescence binding studies confirm ridinilazole binding to DNA.

**FIG 1:**
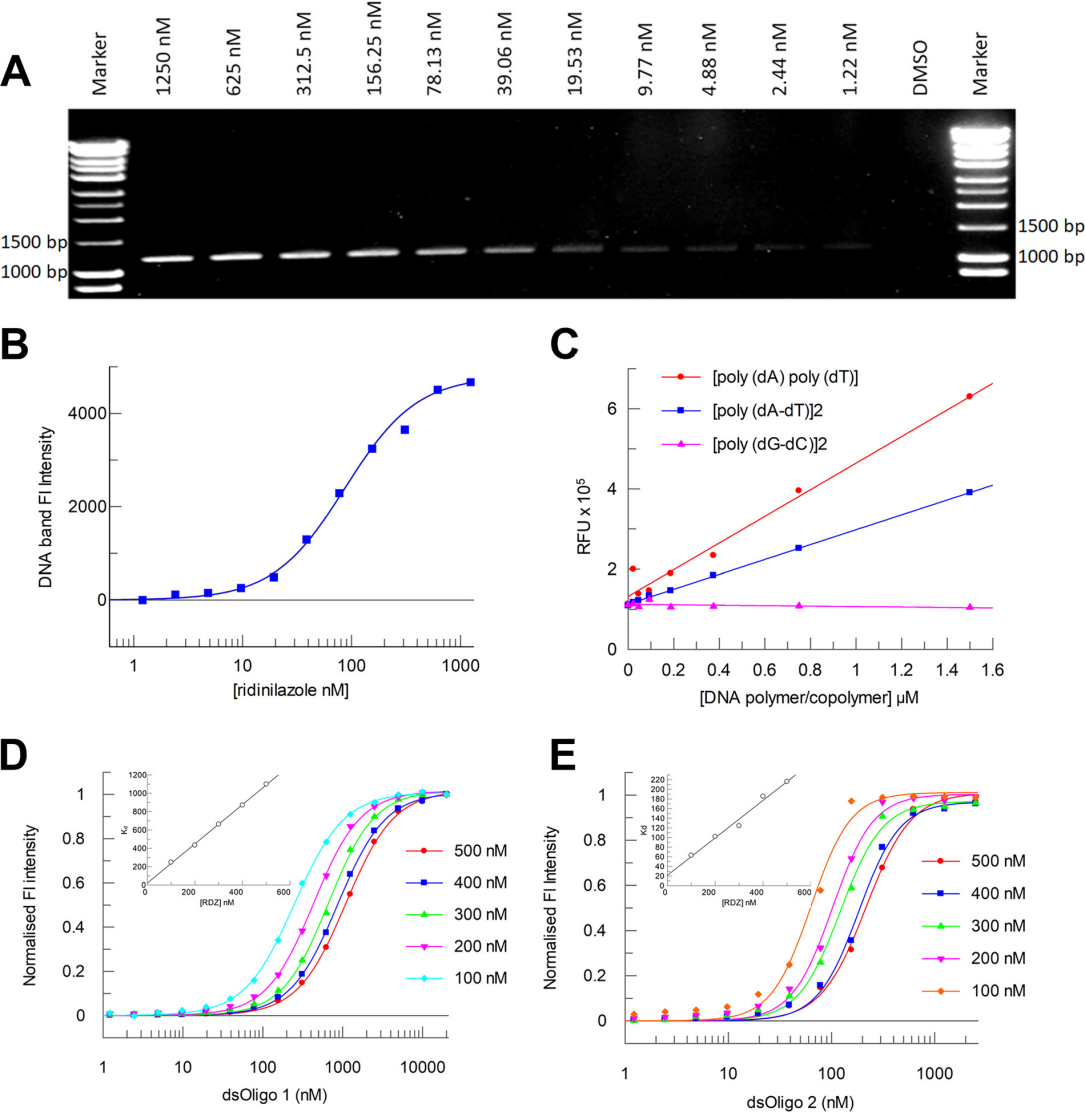
Primary characterization of ridinilazole binding to DNA. (A) UV visualization of ridinilazole (1250 to 1.22 nM) bound to DNA (2 μg C. difficile amplicon). (B) Densitometry plot of the fluorescent bands seen in Fig. 1A, an apparent K_d_ of 87 nM was determined. (C) Titration of DNA polymers with 2 μM ridinilazole. No increase in fluorescence was observed for (poly[dG-dC])_2_ DNA polymer. Enhanced fluorescence was observed with (poly[dA-dT])_2_ and poly(dA)·poly(dT) polymers. (D and E) Titrations of two double-stranded DNA oligonucleotides with fixed concentrations of ridinilazole. Extrapolated dissociation constants in insets yielding K_d_ values of 21.6 nM for dsOligo 1 (CGCGAATTGCGC) and 21.3 nM for dsOligo 2 (CGCAAATTTGCG). Binding data represents mean of three replicates.

To study the potential binding of ridinilazole to DNA, agarose gel electrophoresis experiments were first performed. Separated DNA was visualized directly using the intrinsic fluorescence of ridinilazole which increases >100-fold upon binding to DNA. A ridinilazole titration using a fixed concentration of a C. difficile-derived *metK* amplicon demonstrated ridinilazole-associated fluorescence upon UV transillumination in the absence of any other DNA dye (Fig. 1A). Associated densitometry yielded an apparent K_d_ of <100 nM (Fig. 1B), most likely from multiple binding events across the DNA amplicon. This apparent K_d_ was significantly lower than the ridinilazole MIC_90_ against C. difficile isolates, typically 0.25 μg/mL (9) or ~550 nM. Initial specificity studies revealed a much greater binding specificity for AT-containing double-stranded DNA polymers. At fixed ridinilazole concentrations, a poly-GC polymer (poly[dG-dC])_2_ showed no increase in fluorescence intensity compared to two AT-based polymers, (poly[dA]·poly[dT]) and (poly[dA-dT])_2_ (Fig. 1C). Using large DNA substrates, such as the polymers, could allow multiple binding events; therefore, further biochemical characterization was completed using shorter double-stranded oligos of 12 bp in length. RDZ binding to multiple oligos was confirmed and two double-stranded oligos with an AT core sequence conservation are highlighted in Fig. 1D and E. Due to the sensitivity of the ridinilazole fluorescent readout, tight binding analysis was required to define some of the dissociation constants for some of the more potent ridinilazole:oligo interactions. Using this methodology, K_d_ values as low as 21 nM were recorded for RDZ (Fig. 1D and E), while equivalent assays with Hoechst 33258 returned a K_d_ of 61.6 nM. Collectively, these data provided strong confirmation of DNA binding by ridinilazole.

### DNA footprinting studies confirm ridinilazole AT binding specificity.

**FIG 2:**
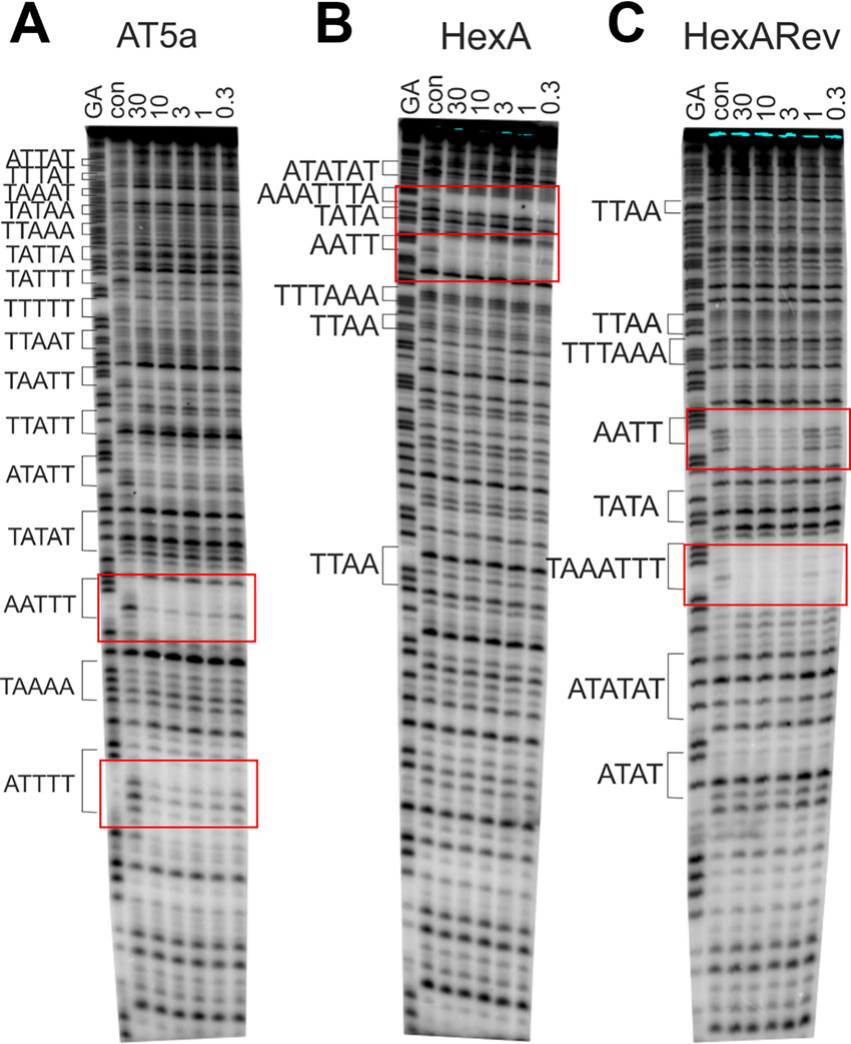
DNase I footprinting patterns for ridinilazole on three DNA AT based templates AT5a (A), HexA (B), and HexARev (C). The ligand concentration (μM) is shown at the top of each gel lane. Tracks labeled “GA” are Maxam-Gilbert markers specific for purines, while “con” indicates (control) cleavage without added ligand. The position of each of the AT sites is indicated in brackets. Sequences located at the top of HexA are toward the bottom of HexARev and *vice versa*. Areas of proposed ridinilazole interaction are boxed in red.

Initial DNA binding studies indicated a ridinilazole binding preference for AT sequences. To probe the DNA binding specificity in more detail, DNase I footprinting was completed using ^32^P-labeled template DNAs. In anticipation that ridinilazole might prefer binding to specific arrangements of AT base pairs, we prepared a DNA sequence that contained all 16 combinations of five consecutive (A or T) base pairs (AT5 sequence template detailed in the Materials and Methods section). We chose to separate these sites with the four base pair sequence CGTG/CACG, to which the ligand was not expected to bind. The results of DNase I footprints with these fragments are shown in Fig. 2. With the AT5a template (Fig. 2A), the clearest footprint could be seen at AATTT, which persisted to concentrations below 1 μM. ATTTT also appeared to be a good binding site. Other regions of weaker attenuated cleavage were evident at ATATT, TAATT, and TTTTT at the highest concentrations, with some hints of protection of other AT sites. An interesting effect could be seen between TATTT and TTTTT on the AT5a template, in which there was enhanced DNase I cleavage in the presence of the drug. Such local regions of enhanced DNase I cleavage are often interpreted as arising from drug-induced changes in local DNA structure that make it a better substrate for cleavage by the enzyme (14). A similar effect can be seen between AAATA (TATTT) and TAATA (TATTA). A second set of DNase I footprinting experiments are detailed using the HexA and HexARev templates in Fig. 2B and C, these together contain 32 symmetrical hexanucleotide sequences, cloned in opposite orientations for each template. Clear footprints could be seen at AAATTTA on HexA and the opposite TAAATTT sequence on HexARev, AATT is also indicated as a good binding sequence being present on both HexA and HexARev. The DNase I footprinting data confirmed a clear ridinilazole preference for binding AT sequences in the target DNA binding site.

### A ridinilazole:DNA complex reveals a minor groove binding mechanism with AT specificity.

**FIG 3:**
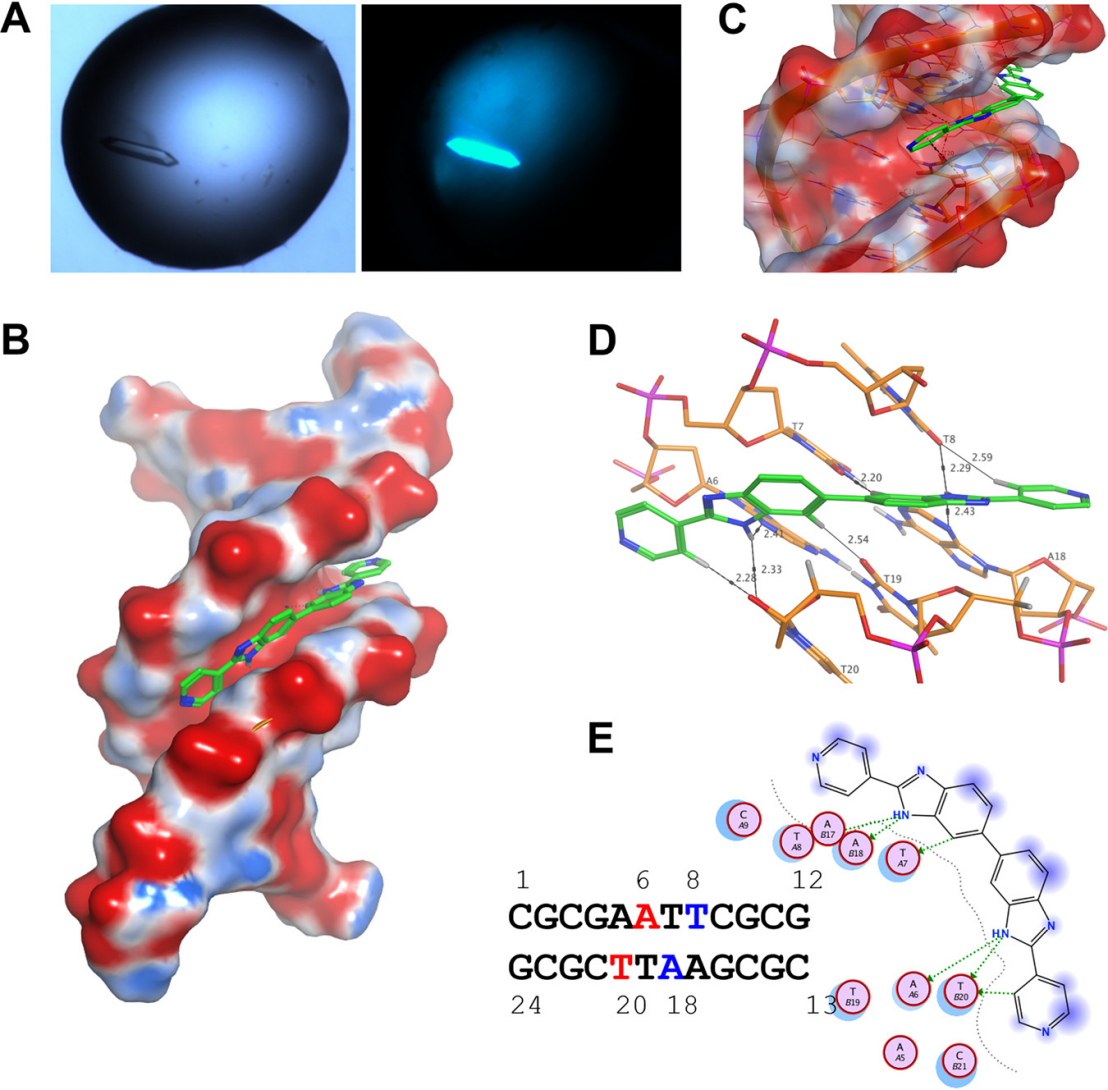
Ridinilazole-DNA structural studies. (A) Co-complex crystals were identified through UV illumination of screen plates; ridinilazole:DNA crystal complexes appeared as intense blue. (B) The resulting ridinilazole complex structure with the antibiotic clearly positioned in the DNA minor groove. (C) Ridinilazole adopts an angled conformation wrapping the dsDNA oligonucleotide. (D) The bonding interactions between ridinilazole and the DNA substrate, each benzimidazole NH H-bonding to separate nonpaired adenine and thymine bases. (E) A two-dimensional (2D) interaction map providing more bonding detail, the nonpaired adenine and thymine bases bonding to each benzimidazole group interaction are highlighted in red or blue, respectively, in the dsOligo1 sequence.

To build on the biochemical understanding of ridinilazole binding to DNA substrates, structural studies were conducted to identify the precise localization of DNA binding. Crystallographic screens were completed with double-stranded oligos, using localization alone and in complex with ridinilazole. The intrinsic fluorescence of ridinilazole was used to identify co-complex crystals through illumination of screening solutions with UV light; all four oligos yielded crystals of varying size and quality. dsOligo 1 (CGCGAATTCGCG) + ridinilazole produced rod-like crystals with blue fluorescence upon UV exposure (Fig. 3A), an X-ray diffraction data set was collected, and a resulting structure was determined to a resolution of 2.2 Å. The structure was solved in P1 by molecular replacement using PDB 3U2N (15) and three co-complex molecules were observed in the asymmetric unit. Unexplained electron density was well described by the modeled ridinilazole molecule, confirming the compound binding location in the DNA minor groove (Fig. 3B). Ridinilazole was shown to adopt a curved snug fit typical of other minor groove binders, wrapping itself around the DNA with a dihedral angle of 34.5° between the two halves of the molecule (Fig. 3C). The model positions the symmetrical bisbenzimidazoles proximal to the core -AATT- of the double-stranded oligo with the bond connecting the symmetrical heterocycles positioned directly above the central -AT- pair (Fig. 3D). The central benzimidazoles both interact via their NH groups with neighboring nonpaired adenine and thymine bases; there are H-bonds from one NH to the adenine moiety of A6 (2.41 Å) and to the thymine moiety of T20 (2.33 Å), and from the other NH to the adenine moiety of A18 (2.43 Å) and the thymine moiety of T8 (2.29 Å) (Fig. 3E). The four-position CH of the benzimidazoles H-bond with T19 (2.54 Å) and T7 (2.2 Å), respectively (Fig. 3E). On each of the terminal pyridyl groups, one 2-position CH interacts with a thymine carbonyl (T20 2.28 Å; T8 2.59 Å). The terminal pyridyl groups of ridinilazole bookend the complex through tight interactions with opposing deoxyribose groups on each strand (Fig. 3C). The overall structural positioning of ridinilazole was unambiguous and identical across the three molecules present in the unit cell. The combined biochemical data confirmed that ridinilazole not only binds DNA but is a minor groove binder with the AT binding specificity confirmed.

### Fluorescence imaging confirms ridinilazole C. difficile genomic localization.

**FIG 4:**
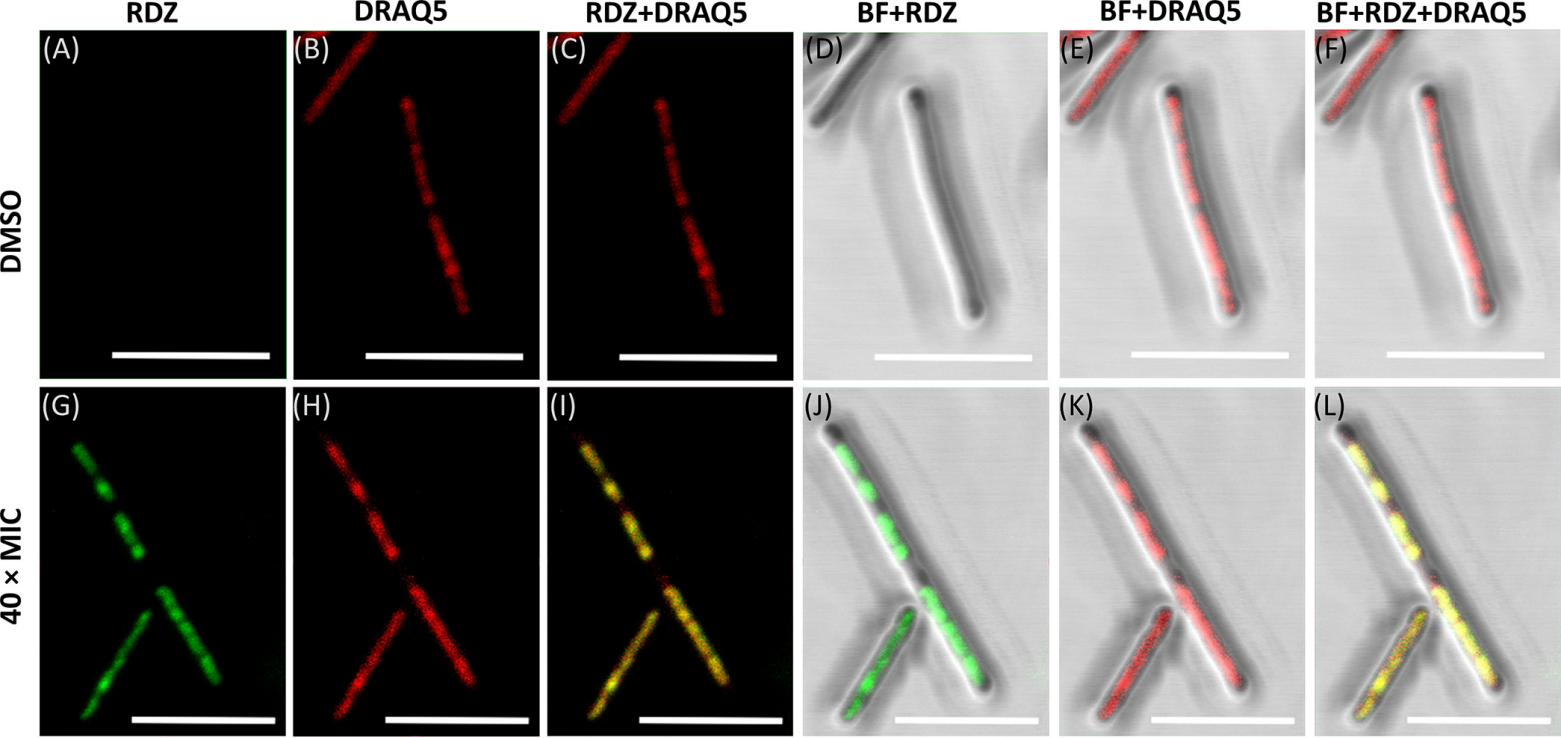
Colocalization analysis of ridinilazole (RDZ) and DRAQ5 fluorescence by confocal laser scanning microscopy in the C. difficile strain 630. Upper and lower panels represent the cells exposed to DMSO (A to F) and 40 × MIC of RDZ (G to L) for 15 min. Cells were stained with DNA dye DRAQ5. RDZ fluorescence (shown in green) and DRAQ5 fluorescence (shown in red) was excited by the violet (405 nm) and red (640 nm) laser, respectively. BF denotes the brightfield image whereas “+” denotes the merging mode of two or three different images. Scale bar, 5 μm.

Ridinilazole cellular localization studies using fluorescence confocal microscopy were used to confirm the DNA binding mechanism of action. These studies utilized the intrinsic fluorescence of ridinilazole to link DNA binding to observed morphological effects of ridinilazole treatment (16). The antibiotic demonstrated very strong spatial co-localization with DRAQ5-stained DNA (Fig. 4G and H and co-localization in Fig. 4I and L) with a mean Pearson correlation coefficient of greater than 0.99 (100 cells analyzed, *P* < 0.0001, two-sided *t* test). Co-localization was specific to RDZ and DRAQ5-stained DNA with no RDZ signal present in the DMSO control samples (Fig. 4A and D). These representative images were obtained following a 15-min exposure to high concentrations of ridinilazole (40× MIC). Similar image profiles were obtained from samples exposed to lower drug concentration (4× MIC) for 15 min or 1 h (Fig. S1 and 2). These supplemental figures also present wider image fields to demonstrate the consistency of staining across numerous C. difficile cells. These studies confirmed that ridinilazole exclusively localizes at the site of genomic DNA in C. difficile cells.

### Deletion of efflux systems in Escherichia coli increases susceptibility to ridinilazole.

**FIG 5:**
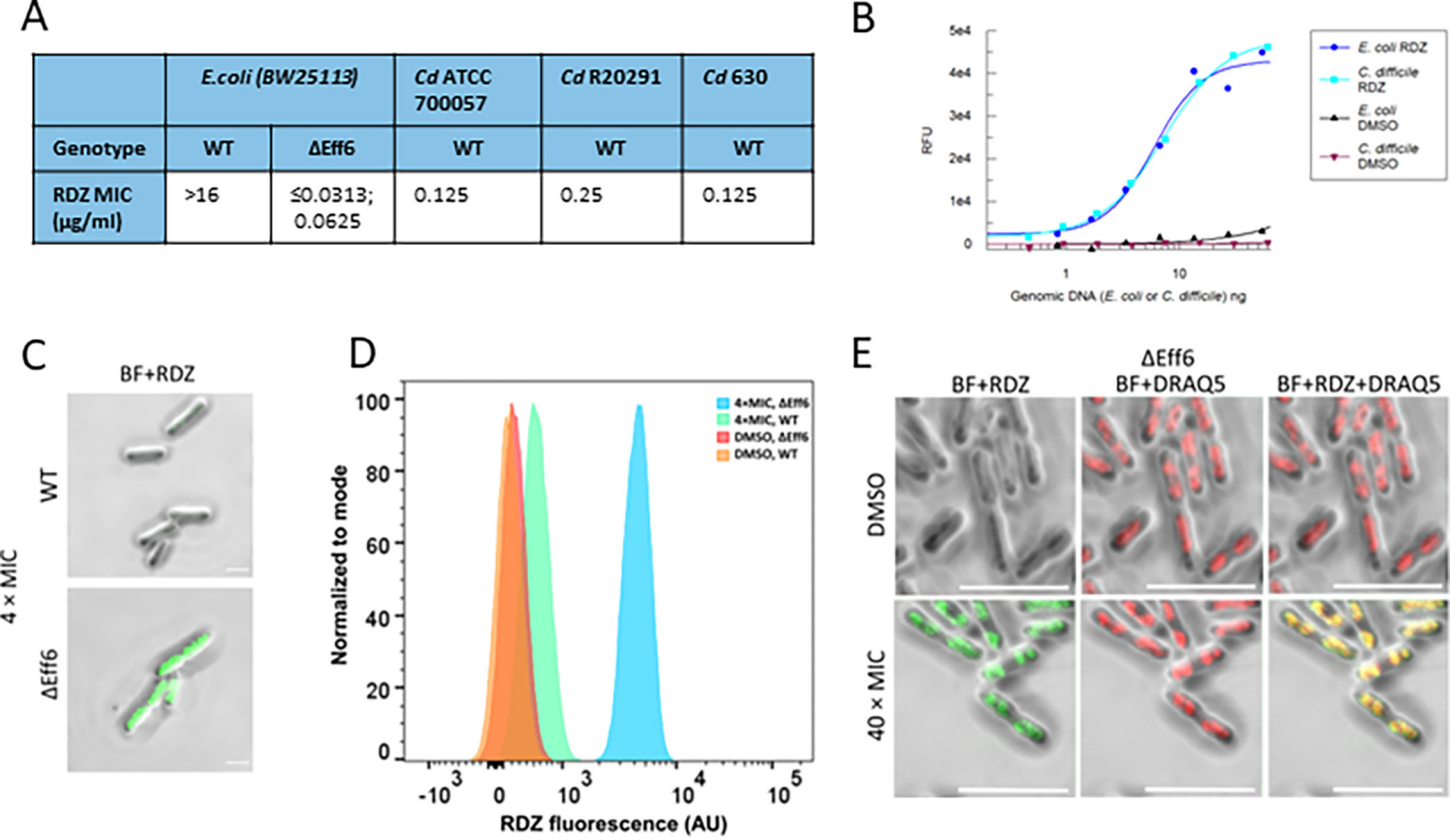
The role of efflux in ridinilazole susceptibility in E. coli. (A) Summary of MIC values of E. coli BW25113 strains (wild-type [WT] and its efflux pump-defective mutant ΔEff6), and C. difficile strains (ATCC700057, R20291, and 630). (B) Binding curves of E. coli and C. difficile genomic DNA with ridinilazole (RDZ), the corresponding DMSO treatment was the control. (C) Confocal laser scanning microscopic images of E. coli BW25113 WT and pump-defective mutant ΔEff6 cells that were exposed to 4 × MIC of RDZ for 2 h. BF+RDZ denotes the composite of brightfield and RDZ fluorescent images. For comparative analysis, the Lookup table (LUT) settings were consistent for both strains. Scale bar, 2 μm. (D) Overlayed flow cytometric histograms showing the contrasted RDZ fluorescence between E. coli BW25113 WT and ΔEff6 that were exposed to 4 × MIC of RDZ for 2 h. The DMSO treatment was the control. Each histogram was a representative one from two independent duplicate experiments for each experimental group. The median RDZ fluorescence for the DMSO-treated WT, DMSO-treated ΔEff6, 4 × MIC-treated WT, 4 × MIC-treated ΔEff6 was 169, 238, 541, and 4325 AU (arbitrary unit), respectively; and the count of the corresponding cell event was 98104, 98477, 29668, and 29674, respectively. (E) Colocalization analysis of RDZ and DRAQ5 fluorescence by confocal laser scanning microscopy in the mutant ΔEff6. Upper and lower panels represent the cells exposed to DMSO and 40 × MIC of RDZ for 15 min. Both DMSO- and RDZ-treated cells were stained with DNA dye DRAQ5. RDZ fluorescence (shown in green) and DRAQ5 fluorescence (shown in red) were excited by the violet (405 nm) and red (640 nm) laser, respectively. BF denotes the brightfield image whereas “+” denotes the merging mode of two or three different images. Scale bar, 5 μm.

E. coli wild-type strains are not susceptible to ridinilazole (17). This could be due to the absence of the drug’s target in E. coli, or a failure of the drug to enter and/or accumulate within the cell. We engineered an E. coli strain (ΔEff6) derived from BW25113 deficient in six multidrug efflux systems. This strain had dramatically increased sensitivity to ridinilazole (Fig. 5A), returning MICs lower than the values observed against C. difficile, while the wild-type BW25113 parental strain was not susceptible. These data complement recent observations that overexpression of an efflux pump (*mdtEF*) restored ridinilazole resistance to a permeable E. coli strain (18). We also demonstrated that the enhanced fluorescence of ridinilazole upon DNA binding *in vitro* was apparent, and comparable when using either E. coli or C. difficile genomic DNA (Fig. 5B). These data argue against the narrow antimicrobial spectrum of ridinilazole being determined by specific and selective binding to C. difficile DNA. The inferred contribution of efflux in restricting intracellular accumulation of ridinilazole in E. coli was exemplified through confocal imaging (Fig. 5C) and flow cytometry analysis (Fig. 5D). Wild-type BW25113 and ΔEff6 cells were exposed to high concentrations of ridinilazole (equivalent to 4× C. difficile MIC, 2 h) to reveal the intracellular staining differential across these strains in these two analyses. These data reveal increased intracellular levels of ridinilazole (by virtue of enhanced fluorescence upon binding DNA) in the efflux-deficient background (ΔEff6) compared to the wild-type strain (BW25113). We propose that the accumulation of ridinilazole in the efflux-compromised strain (ΔEff6) results in elevated intracellular concentrations of drug coincident with the pharmacological (antimicrobial) response, as evidenced in the reduced MIC observed against this strain (compared to wild-type). We also demonstrated, through overlay of RDZ fluorescence with DRAQ5 fluorescence, that ridinilazole co-localizes to genomic DNA within E. coli (Fig. 5E). The RDZ staining pattern in the E. coli mutant is similar to that observed in C. difficile cells.

### Ridinilazole induces pleiotropic transcriptional dysregulation.

With a bacterial cell target identified and confirmed, we next probed the mechanistic consequences of ridinilazole binding to DNA in a cellular context using RNA-seq to begin to determine how ridinilazole exerts its antimicrobial activity on C. difficile. We measured transcriptomic changes in C. difficile strain 630 upon exposure to ridinilazole at 4× MIC compared to DMSO control. The immediate transcriptional response was determined at 15-min postexposure, followed by later hourly time points up to 3 h; four replicates were used for each treatment time point.

**FIG 6:**
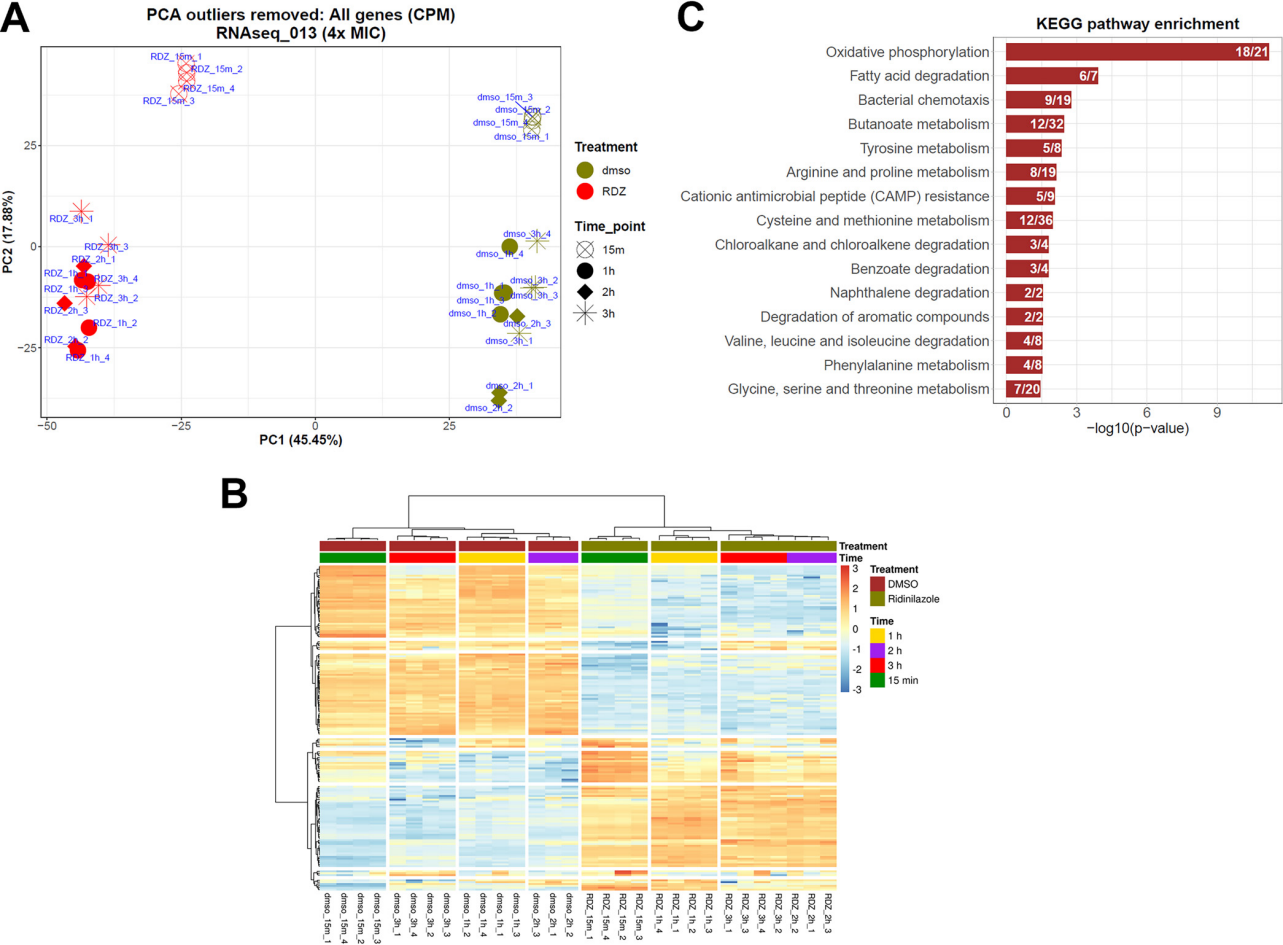
Transcriptional changes induced by Ridinilazole. (A) Scatterplot of Principal Component Analysis scores from all genes in C. difficile strain 630 treated with 4× MIC ridinilazole (0.25 μg/mL) or DMSO at 15 min, 1 h, 2 h, and 3 h postexposure. Principal Components 1 and 2 together accounted for 63.33% of variance in the data set. (B) Heatmap of 163 genes (top 100 differentially expressed genes and the genes involved in oxidative phosphorylation, bacterial chemotaxis, cationic antimicrobial peptide [CAMP] resistance, and Stickland metabolism pathways). The rows represent genes and the columns represent samples from all four time points. A high-resolution copy (Supplementary Data File 2) of this heatmap with gene names has been made available through GitHub (see Data Availability section). The genes are also listed in Table S6. (C) KEGG pathway enrichment at 15 min time-point. The bars represent the negative logarithm (base 10) of the enrichment *P*-values. The numbers inside the bars represent the ratio of the number of differentially expressed genes in the query set and the total number of genes in the pathway.

We examined the general pattern of gene expression changes using a principal component analysis (PCA). PCA showed global changes in gene expression (Fig. 6A; Fig. S3). Transcriptional changes (ridinilazole versus DMSO) were established at 15-min postexposure, and the pattern remained the same at all time points (Fig. 6A and B; Supplementary Data File 1). We focused further analysis on the 15-min time-point, to allow for immediate exploration of transcriptional changes in C. difficile before compensatory mechanisms could further complicate direct ridinilazole transcriptional effects. Upregulation of 325 genes and downregulation of 407 genes was observed at the 15-min time-point (Table 1). Of the top 60 upregulated genes, eight comprise the entirety of the glycine reductase operon (*grdX*, *trxB3*, *trxA2*, *grdEABCD*) (Table S1) (17). Glycine reductase is a key enzyme in the Stickland pathway which couples the oxidation and reduction of amino acid pairs to the formation of ATP and is the major anaerobic mechanism by which C. difficile generates energy (18). The most efficient Stickland electron acceptors are glycine, proline and hydroxyproline (17) and we observed that genes in the proline reductase operon were among the most downregulated genes (*prdABDE prdE2 prdF*). Inspection of other genes in the Stickland pathway (19, 20) (Fig. 6B; Table S1) showed that in addition to the changes in glycine and proline reductase mRNA levels, aldehyde-alcohol dehydrogenase (*adhE* and *adhE1*) were upregulated, and phosphate butyryltransferase (*ptb*) downregulated by ~8-fold.

**TABLE 1:** Number of differentially expressed genes in CD630 between treatment groups (DMSO and Ridinilazole [4× MIC]) at the four different time points (15 min, 1 h, 2 h, 3 h)

Contrasts	No. of differentially expressed genes (abs[log2FoldChange] > 2 & padj < 0.01)	Percentage of differentially expressed genes (vs total no. of genes in C. difficile)	No. of up-regulated genes	No. of downregulated genes
Ridinilazole vs DMSO at 15 min	732	19.62%	325	407
Ridinilazole vs DMSO at 1 h	940	25.20%	415	525
Ridinilazole vs DMSO at 2 h	983	26.35%	462	521
Ridinilazole vs DMSO at 3 h	1021	27.37%	461	560

KEGG pathway analysis implicated 15 pathways were enriched (*P* < 0.05, two-tailed hypergeometric test) in the transcriptional response to 15 min ridinilazole exposure (Fig. 6C; Table S3), with six of these being involved in amino acid metabolism—again possibly impacting Stickland fermentation. Strikingly, in the oxidative phosphorylation pathway (*P* < 0.0001) all the genes encoding the ATP synthase were downregulated (Fig. 6B; Table S2). The ATP synthase uses proton motive force (PMF) to generate ATP. Proline reductase, also downregulated, is coupled to PMF generation by an uncharacterized mechanism (19). This combination of downregulation could further decrease the ability of the cell to generate energy. Also notable was upregulation of eight genes involved in the chemotaxis regulatory network (Table S4). There was no evidence for a ridinilazole-induced alteration in flagella gene expression. Intriguingly, in the cationic antimicrobial peptide (CAMP) resistance pathway, there was downregulation of the *dltDABC* operon which encodes d-alanine transferase (Table S5), an enzyme that adds d-alanine to the cell wall to result in a positive charge on the cell surface.

While these are some examples of affected pathways, the transcriptomic studies as a whole revealed that the impacts of ridinilazole on gene transcription were of quick onset, pleiotropic/pan-genomic in effect, and of prolonged action.

## DISCUSSION

Determination of antibiotic mechanism of action is usually straightforward for target-based discovery but is more complex when compounds have been identified through phenotypic screening. The bisbenzimidazoles have generated interest from a drug discovery perspective for over three decades, and while examples such as the Hoechst dyes have proven to be invaluable cell biology tools, no drugs from this class have yet been approved for any indication. The DNA minor groove binding potential of bisbenzimidizoles has been previously reported and exemplified through structural approaches (20). The antimicrobial properties of the symmetrical head-to-head bisbenzimidazoles (as opposed to the head-to-tail arrangement in Hoechst dyes) were noted nearly 20 years ago, particularly their ability to kill Gram-positive bacteria such as Staphylococcus aureus and Mycobacterium tuberculosis (21). However, these phenotypic bactericidal activities were never reconciled with a defined mechanism. Head-to-head bisbenzimidazoles such as ridinilazole had been noted as potential DNA gyrase and topisomerase inhibitors in bacteria (21). Despite successful clinical progression to phase 3, the precise mechanism of action of ridinilazole has thus far proved elusive.

The new approach undertaken in these studies utilized an as yet under exploited property of ridinilazole, its intrinsic fluorescence. Using a range of different biochemical techniques, the binding of ridinilazole to increasingly defined and selective dsDNA substrates was established, the dissociation constants determined were >25-fold lower than the established ridinilazole MIC_90_ for C. difficile isolates. The observed DNA footprinting sites for ridinilazole correspond to the best sites seen with Hoechst 33258 (H33258) and are generally in AT-tracts that lack a TpA step. However, whereas Hoechst eventually binds to most other (AT)_n_ sites at higher concentrations, we did not observe interaction of ridinilazole with all other (AT)_n_ sites within the range of concentrations that were employed. Most ridinilazole sequences identified in the footprinting contained a conserved AATT sequence which was coincident in the two oligonucleotides with greatest binding affinity and within the double-stranded oligo that yielded the ridinilazole:DNA structure. We therefore consider that ridinilazole shows greater discrimination between different arrangements of AT-residues than H33258, possibly due to the differential arrangement of benzimidazole groups in the two compounds. That specific positioning of ridinilazole within the DNA minor groove was comprehensively determined via the co-complex crystal structure, where the molecule was seen to adopt the classic crescent shape of other minor groove binders (20).

Ridinilazole-DNA binding resulted in broad transcriptional perturbation, with over 19% of the transcriptome modified negatively or positively by the antibiotic within 15 min and continued to the study endpoint at 3 h. Of note was the very significant impact on genes involved in energy generation via the Stickland pathway and ATP production via ATP synthase. Perturbation of these important pathways may reduce the ability of ridinilazole-treated C. difficile cells to efficiently generate ATP for the main energy requirements of the bacterium. Changes to cellular energy production might be expected to affect multiple compartments, including cell division, and depletion of energy reserves may explain ridinilazole’s initial bacteriostatic effect of >8 h before cell death (8). Further studies will be required to assess which transcriptional changes correlate most closely with ridinilazole’s bactericidal effects. Of interest were the transcriptomic changes (downregulation of *dltDABC* operon) to the protective CAMP pathway in C. difficile. While not involved directly in the *in vitro* bactericidal effects observed, perturbation of this pathway may indicate decreased ability to defend against host-derived antimicrobial peptides *in vivo*. The addition of d-alanine to the cell wall via d-alanine transferase results in a positive charge on the cell surface, decreasing the ability of CAMPs to interact with Gram-positive bacteria, including C. difficile (22). If transcription of the *dltDABC* operon is also affected *in vivo*, it is possible that this could result in an increased sensitivity to host CAMPs during infection.

In conclusion, this study has elucidated the primary mechanism of action of ridinilazole as a DNA minor groove binder antibiotic. Intracellular accumulation of drug to the DNA minor groove allows DNA binding, transcription dysregulation, and eventual cell death even if DNA replication is initially unperturbed by ridinilazole. The selectivity of ridinilazole for its very narrow range of bacterial targets will require further study. Intracellular accumulation of drug to levels sufficient to exert its effect will be an outcome of the balance of permeation and efflux. Indeed, we present data showing that efflux-deficient E. coli become susceptible to ridinilazole. It is likely that the drug’s narrow spectrum of activity is driven at least in part by being a substrate for efflux systems in other species. Such a new mechanism represents an exciting development in the field of antibiotic research, where the dearth of new mechanism antibiotics has challenged the ability to effectively treat AMR infection. The data presented here demonstrates that broad disruption of gene expression represents a viable new approach for antibiotic discovery and development. Minor groove binding antibiotics promise to be significant new additions to the antimicrobial armamentarium. Most importantly, however, is the potential for ridinilazole to treat CDI patients, where recurrence remains a serious unmet medical need for patients.

## MATERIALS AND METHODS

### Bacterial strains and MIC determination.

Representative laboratory and wild-type C. difficile strains CD630, R20291, and ATCC700057 were used in the studies. E. coli BW25113 was used as a wild-type representative and the six-pump knockout (ΔEff6) was generated in BW25113 by sequential suicide plasmid mediated allelic exchange of *acrAB*, *acrD*, *emrAB*, *macAB*, *mdfA*, and *mdtK*. MICs of ridinilazole against E. coli BW25113 (wild-type) and ΔEff6 were determined by aerobic broth microdilution in cation-adjusted Mueller-Hinton Broth (CA-MHB). MICs for C. difficile were determined anaerobically on supplemented brucella blood (SSB) agar.

### DNA amplicon visualization.

A C. difficile *metK* (S-adenosylmethionine synthetase) gene amplicon of 1,194 bp was generated by PCR from 5 ng of strain 630 genomic DNA using primers ATGGCAAGACATTTATTTACGTCAGA and TTATTCACCTAAAGCATCTTTTCTTAACTG. Then, 2 μg of the amplicon product was incubated with a 2-fold dilution series of ridinilazole for 10 min before separation of the amplicon:label mix alongside 1 kb DNA markers (incubated with 10 μM ridinilazole) on a 1% agarose gel in TBE. Ridinilazole concentrations in Fig. 1A represent the final concentration in loading buffer. Excitation energy was provided via a UV transilluminator and fluorescence recorded using a Syngene imaging system camera.

### Determination of DNA sequence specificity.

To test the binding specificity of ridinilazole, three polymers, that are double-stranded DNA models used for conformational studies of DNA structure and drug interactions, were titrated with a fixed concentration of ridinilazole of 2 μM. Polymers used were sourced from Sigma-Aldrich. These were Poly(deoxyguanylic-deoxycytidylic) acid sodium salt (Sigma cat no. P9389-10UN) (poly[dG-dC])_2_, Polydeoxyadenylic acid • Polythymidylic acid sodium salt (Sigma cat no. P9764-5UN) (polydA·poly dT) and Poly(deoxyadenylic-thymidylic) acid sodium salt (Sigma cat no. P0833-10UN) (poly[dA-dT])_2_. Polymers were resuspended in buffer containing 50 mM Tris-HCl, 100 mM NaCl and 0.1 mM EDTA, pH 7.4. Polymer binding studies were performed in 200 μL assay volumes in Greiner black, flat-bottom 96-well plates (Catalog number 655096). Polymers and ridinilazole were equilibrated in plates for 15 min before being read on a CLARIOstar Plus at excitation wavelength of 355+/−20nm - see equivalent text in next section ‘determination of dissociation constants’ and emission of 455+/−30nm - see equivalent text in next section ‘determination of dissociation constants’. Data analysis was performed with Grafit (Erithacus Software Limited), using a linear fit, y = a + bx.

### Determination of dissociation constants.

Oligonucleotides were provided by Integrated DNA Technologies and Eurofins. Oligos were either annealed or self-annealed as appropriate on a thermal cycler. The block temperature was ramped to 95°C and then cooled at 0.1°C/s to 20°C. Ridinilazole and oligo binding studies were performed in 20 μL assay volumes in Greiner black, flat-bottom, low volume, Hibase 384-well plates (Greiner 784076-25), in a buffer containing 50 mM Tris-HCl, 100 mM NaCl, and 0.1 mM EDTA, pH 7.4. Oligos were titrated before addition of a fixed concentration of ridinilazole across the series, plates were equilibrated for 10 min before being read on a CLARIOstar Plus (BMG Labtech) at excitation wavelength of 355 ± 20 nm and emission of 455 ± 30 nm. Data analysis was performed with Grafit (Erithacus Software Limited), using a cooperative fit equation y = ([L]^N^ × Cap.)/(K^N^ + [L]^N^), where L = ligand concentration, Cap = Capacity (−intercept/gradient), K = K (−1/gradient) and N = Cooperativity. Where required, extrapolated tight binding K_d_ values were determined by linear fit, y = a + bx.

### DNase I footprinting.

Plasmids containing synthetic DNA inserts were constructed by cloning the inserts into the BamHI site of pUC18 or pUC19 and were maintained in E. coli TG2. Fragments AT5a and AT5b were prepared in a similar way and contained all 16 arrangements of (A/T)_5_ each separated by the sequence CGTG/CACG, to which the ligand was not expected to bind. AT5a and AT5b contained the same sequence, but were cloned in opposite orientations. Radiolabelled DNA fragments containing these inserts were obtained by cutting the plasmids with HindIII and SacI, and were labeled at the 3′-end of the HindIII site with α-^32^P[dATP] using Klenow fragment (exo-). The labeled fragments of interest were separated from the rest of the plasmid DNA on 6% nondenaturing acrylamide gels, eluted and resuspended in 10 mM Tris-HCl pH 7.4 containing 0.1 mM EDTA to give about 10 c.p.s/μL as determined on a hand-held Geiger counter (approximately 10 nM fragment). DNase I footprinting was performed as previously described (23, 24). Briefly, 1.5 μL of radiolabeled DNA was mixed with 1.5 μL of appropriately diluted ligand and incubated at 37°C for 1 to 30 min. The complexes were digested with 2 μL DNase I (final concentration about 0.02 units/mL) that had been diluted in 20 mM NaCl, 2 mM MgCl_2_, 2 mM MnCl_2_, and the reaction was stopped after 1 min by adding 5 μL of formamide containing 10 mM EDTA, 1 mM NaOH, and 0.1% bromophenol blue. The products of the reaction were heated at 100°C for 3 min, crash cooled on ice, and separated on 8% denaturing polyacrylamide gels containing 8M urea. The gels were fixed in 10% acetic acid, transferred to Whatmann 3MM paper and exposed to a storage phosphor screen overnight before scanning with a Typhoon phosphorimager. Complexes used:

**HexA and HexARev**: 5′-GGATCCCGGGATATCGATATATGGCGCCAAATTTAGCTATAGATCTAGAATTCCGGACCGCGGTTTAAACGTTAACCGGTACCTAGGCCTGCAGCTGCGCATGCTAGCGCTTAAGTACTAGTGCACG TGGCCATGGATCC-3′

**AT5a and b:** 5′-GATCCGTGAAAATCGTGTTTTACGTGAAATTCGTGATATACGTGAATATCGTGAATAACGTGAATTACGTGATTAACGTGAAAAACGTGAAATACGTGTAATACGTGTTTAACGTGTTATACGTGATTTACGTGATAAACGTGATAATCGTGGATC-3′

### Structure studies.

Lyophilized HPLC purified ssDNA was reconstituted in distilled water at a concentration of 20 mM and stored at 4°C for up to 4 months. Prior to crystallization, ssDNA stocks were annealed using a thermocycler. Annealing was performed by heating ssDNA for 5 min at 95°C followed by rapid cooling to 4°C over 1 min. Prior to crystallization trials, annealed oligonucleotides were characterized in the fluorescent binding assay to confirm that annealed oligonucleotides displayed ridinilazole binding. Crystallization trials were performed using dsDNA oligonucleotides alone and in complex with drug. Four dsDNA oligos were screened:

CGCGAATTCGCG – self annealed to form dsDNA
CGCAAATTTGCG − self annealed to form dsDNA
CGTCTTTATTCA and TGAATAAAGACG – annealed together
CATATTTATTTC and GAAATAAATATG – annealed together

The dsDNA+ridinilazole stock solutions were made by adding compound, dissolved in DMSO, to dsDNA at a 1:1 molar ratio. dsDNA+ridinilazole complex solutions had a final DMSO concentration of 4%. Solutions were incubated for 15 min at 20°C. After incubation, solutions were clarified by centrifugation at 17,000 *g* for 5 min to remove compound precipitant. The dsDNA+ridinilazole solutions were then used for crystallization screening. Sparse matrix screens HELIX and Structure Screen 1 + 2 were used for initial crystallization screening. Screening was performed with 1 mM dsDNA or 1 mM dsDNA+ridinilazole solutions using 1:3, 1:1, and 3:1 dsDNA:reservoir buffer ratios. For initial screening, the crystallization drop volume was 0.2 μL and the reservoir volume was 40 μL. For optimization, the crystallization drop volume was 1 μL and the reservoir volume was 140 μL. Crystallization plates were stored at 14°C and crystals formed between 1 and 7 days. Selected crystallization hits were optimized through variation in buffer components. Crystallization plates were imaged using a SpectroLight 610 (XtalConcepts GmbH) and a XtalLight 100 UV module with a 385 nm short pass filter. Crystals selected for data collection were mounted into loops and flash-cooled in liquid nitrogen without cryoprotectant. X-ray diffraction data collection for the DNA crystals was conducted on Diamond Light Source (Didcot, Oxford, UK) beamline I04 fitted with an Eiger2 × 16M detector (Dectris). X-ray diffraction data were indexed and integrated using DIALS (25). Data were scaled and merged using AIMLESS where resolution cut-offs were applied. The structures were solved by molecular replacement using Phaser (26) and PDB 3U2N (15). AceDRG was used to create ligand restraints for ridinilazole and programs Refmac5 and Coot were used for refinement of the structures. Validation of the structures was achieved using PDB validation. All structural images and superpositions were generated in CCP4mg.

### Confocal microscopy studies.

C. difficile cells were grown in a vinyl anaerobic chamber (Coy Lab Products) at 37°C using brain heart infusion-supplemented (BHIS, Hardy Diagnostics) supplemented with 0.1% sodium taurocholate (Alfa Aesar) and 1% oxyrase enzyme (Oxyrase, Inc.). C. difficile 630 stock was streaked onto CCFA agar and incubated overnight. A single colony was selected and inoculated into BHIS broth and incubated for 16 h. The culture was then used to inoculate BHIS broth at a 1:100 dilution and grown to an optical density at 600 nm of 0.4. Assay cultures were then made by diluting 1:10 into BHIS broth containing DMSO 1% (control group) or ridinilazole (RDZ, final concentrations: 4 × MIC and 40×MIC). Cultures were incubated for 15 min or 1 h, harvested by centrifuging at 3,082 *g* for 10 min and fixed with 4% paraformaldehyde at room temperature for 1 h. After washing, the cells were stained with DRAQ5 (Novus Biologicals, Cat# NBP2-81125, 100 μM) at room temperature for 30 min. After further washing, the cells were resuspended in PBS, spread onto poly-L-lysine coated glass slides, and allowed to air dry for 1 h. Dried cells were mounted with VECTASHIELD HardSet Antifade Mounting Medium (Vector Laboratories, Cat# H-1400-10).

E. coli cells were streaked onto blood agar and incubated at 37°C for overnight. A single colony was inoculated into 10 mL BHI broth and incubated at 37°C for 16 h. The grown liquid cultures were inoculated into the BHI broth with the 1:100 dilution and incubated at 37°C. When the optical density of the culture at 600 nm reached 0.4, the culture was inoculated with 1:10 dilution separately into the BHI broth containing DMSO (1%, control group), or RDZ at the same concentrations as C. difficile experiments above. Cultures were incubated for certain experimental periods (15 min and 2 h). The control and RDZ-treated E. coli cells subsequently underwent a series of steps (harvesting, fixing, staining with DRAQ5, and slide preparation) as described above for C. difficile cells.

Confocal imaging analysis of these C. difficile and E. coli cells was performed with an Olympus FV3000 Inverted Confocal Microscope (Olympus, Japan) using a 60× oil objective. Laser wavelengths of 405 nm and 640 nm were used to visualize the staining of RDZ and DRAQ5, respectively.

### Flow cytometry.

E. coli BW25113 (wild-type) and its efflux pump-defective mutant (ΔEff6) stocks were streaked onto blood agar plates and incubated at 37°C overnight. A single colony for each strain was inoculated into BHI broth and incubated overnight at 37°C. Cultures were then diluted 100-fold into BHI broth and incubated at 37°C. When the optical density (600 nm) of the culture reached 0.4, the culture was further diluted by 10-fold into BHI broth. RDZ was added to each culture at a final concentration of 4 × MIC or equal volume of DMSO (control). The culture was incubated for 2 h at 37°C, harvested by centrifuging at 3,082 g for 10 min, and washed with PBS. Subsequently, cells were fixed with 4% paraformaldehyde at room temperature for 1 h, washed with PBS, and resuspended into PBS. Experimental control and RDZ-treated E. coli cells were analyzed on the BD LSR Fortessa Flow Cytometer equipped with a small particle forward scatter diode assembly. The fluorescence of RDZ was excited by the UV laser (355 nm) and detected by the corresponding filter (450/50 BP). The gating strategy for the single-cell population is described in the supplementary information (Fig. S4).

### RNA-seq/transcriptomics.

RNA was prepared from exponential-phase bacteria treated with 4× MIC ridinilazole (0.25 μg/mL) or DMSO control at 15 min, 1 h, 2 h, and 3 h postexposure. Final solvent concentration was 1% and there were quadruplicate cultures for each time-point. C. difficile 630 was grown overnight (16 h) in BHIS + 0.1% l-cysteine + 0.1% sodium taurocholate under anaerobic conditions in an A45 Workstation (Don Whitley Scientific) and subcultured 1 in 100 into BHIS + 0.1% l-cysteine. At A600nm ~0.4 (5 h to 6 h growth), the culture was diluted 1 in 10 to initiate quadruplicate assay cultures for each condition and time point containing ridinilazole or DMSO, final volume was 40 mL. At each time-point, bacteria were harvested by centrifugation at 4,100 *g* for 5 min at room temperature, resuspended in 1 mL RNAprotect (Qiagen), incubated for 5 min at room temperature and pelleted. Supernatant was removed and the pellets stored at −80°C prior to RNA extraction using an RNeasy minikit (Qiagen) as directed by manufacturer. Mechanical homogenization of cells was by shaking with 0.5 mm Zirconia/Silica beads (Thistle Scientific) at 2,000 rpm. for 2 min (Eppendorf Mixmate). Genomic DNA was removed with TURBO DNase (Thermo Fisher Scientific) and RNA quantified and integrity-checked with a 4200 TapeStation (Agilent). rRNA was removed from 100 ng total RNA for each sample with the Ribo-Zero rRNA Removal Kit (Illumina), cDNA made using the TruSeq Stranded mRNA Library Prep (Illumina), normalized, and pooled prior to sequencing using an Illumina NextSeq 550 sequencer with a single-end 76 cycle run.

The RNA-seq data analysis was performed on a Linux (v5.4.0-88-generic) server with the Ubuntu (20.04.3 LTS) operating system. The data quality was examined using FastQC (v0.11.9) (27) and FastQ Screen (v0.14.1) (28). Quality and adapter trimming was performed using Trim Galore (v 0.6.6; Cutadapt v3.4; Python v3.9.2) (29). Reads were aligned to the C. difficile 630 genome (assembly name: ASM920v2; downloaded from NCBI Reference Sequence [RefSeq]) (30) using Bowtie 2 (v 2.4.2) (31). Alignment quality was examined using Picard tools (v2.25.0) (32) and samtools (v1.12-2-gd515e1b) (33). Aligned reads were assigned to genes using Subread featureCounts (v2.0.2) (34).

The RNA-seq count table was imported into R (v4.0.4) (35) and normalized by trimmed mean of M values (TMM) and transformed to log_2_ count-per-million (CPM) using edgeR (v3.32.1) (36). PCA was performed using the top 500 variable genes or all genes in the genome and two outlier samples (RDZ_2h_4 and dmso_2h_4) were removed. Differential expression analysis was performed using DESeq2 (v1.30.1) (37). Enrichment of KEGG pathways (38) in differentially expressed genes were analyzed using limma (v3.46.0) (39) and KEGG pathway maps were generated using pathview (v1.30.1) (40). Expression of 163 genes that are involved in oxidative phosphorylation, bacterial chemotaxis, cationic antimicrobial peptide resistance, and Stickland metabolism pathways, and the top 100 differentially expressed genes (based on fold change at 15 min; padj < 0.01) were visualized in a heatmap generated using pheatmap (v1.0.12) (41).

### Data availability.

The transcriptomic sequencing data have been deposited and is available at SRA under the project reference PRJNA838023. The data for DNA:ligand structural determination is available in wwPDB under the accession code 7Z9P. The high-resolution version of Fig. 6B can be accessed at: https://summit-therapeutics.github.io/images/RDZ_MOA_Supplementary_Data_File_2.tif.
