# Supplementary material for: The Novel DNA Binding Mechanism of Ridinilazole, a Precision Clostridiodes difficile Antibiotic
Source: Antimicrob Agents Chemother. 2023 Apr 24;67(5):e01563-22. doi: 10.1128/aac.01563-22 (PMC10246881; doi:10.1128/aac.01563-22)
Supplement: Supplemental file 1 — Supplemental material. Download aac.01563-22-s0001.pdf, PDF file, 0.8 MB [file aac.01563-22-s0001.pdf]

The novel DNA binding mechanism of ridinilazole, a precision *Clostridiodes difficile* antibiotic

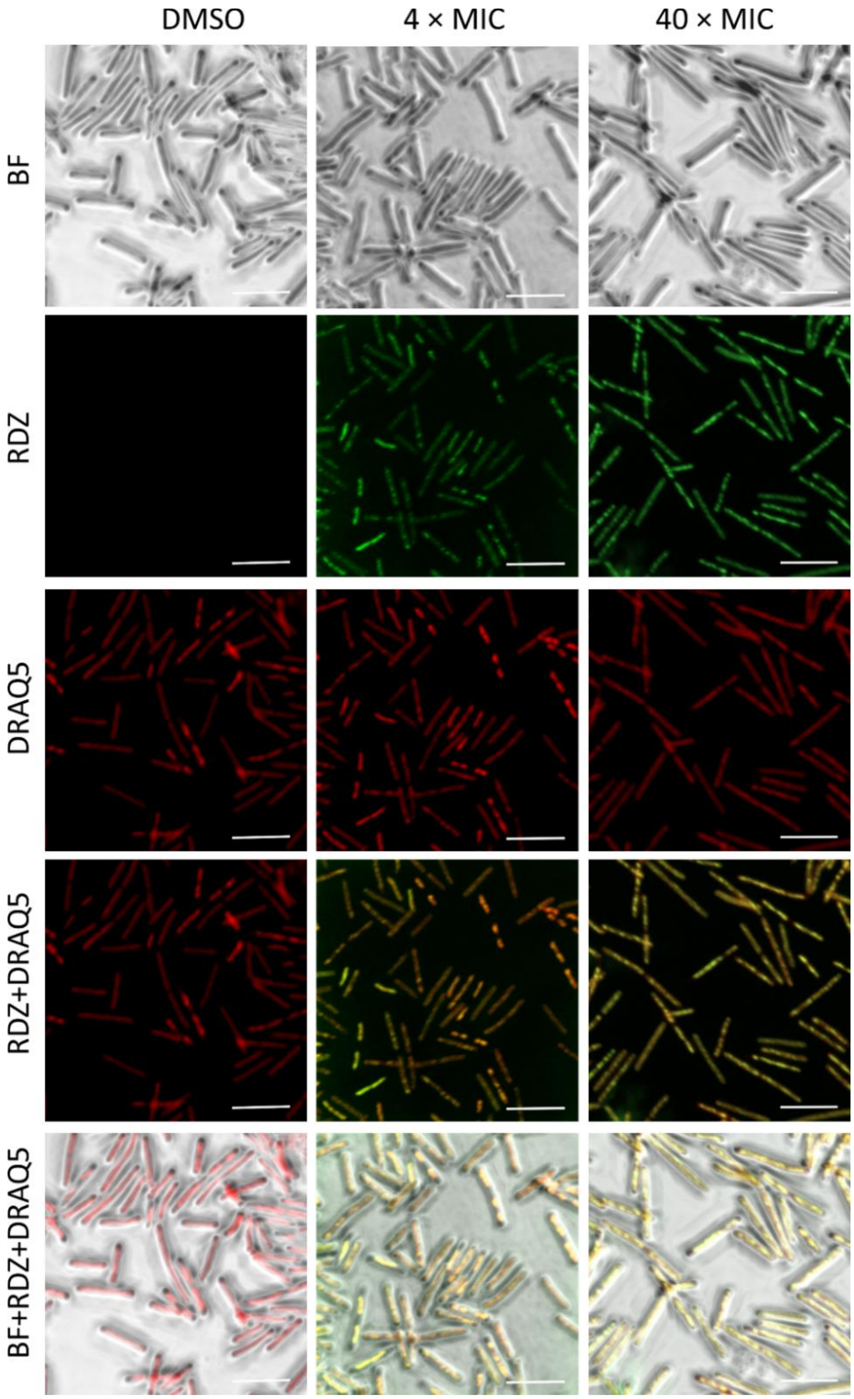

Supplementary Figure 1: Colocalization analysis of ridilinazole (RDZ) and DRAQ5 fluorescence by confocal laser scanning microscopy in the *C. difficile* strain 630. Left, middle, and right panels represent the cells exposed to DMSO, 4 × MIC, 40 × MIC of RDZ for 15 min, respectively. All the cells were stained with DNA dye DRAQ5. RDZ fluorescence (shown in green) and DRAQ5 fluorescence (shown in red) were excited by the violet (405nm) and red laser (640 nm), respectively. The fluorescence shown here is not for quantitative analysis purpose since the Lookup Table (LUT) settings were unstandardized among experimental groups. BF denotes the brightfield image whereas "+" denotes the merging mode of two or three different images. Scale bar, 5 μm.

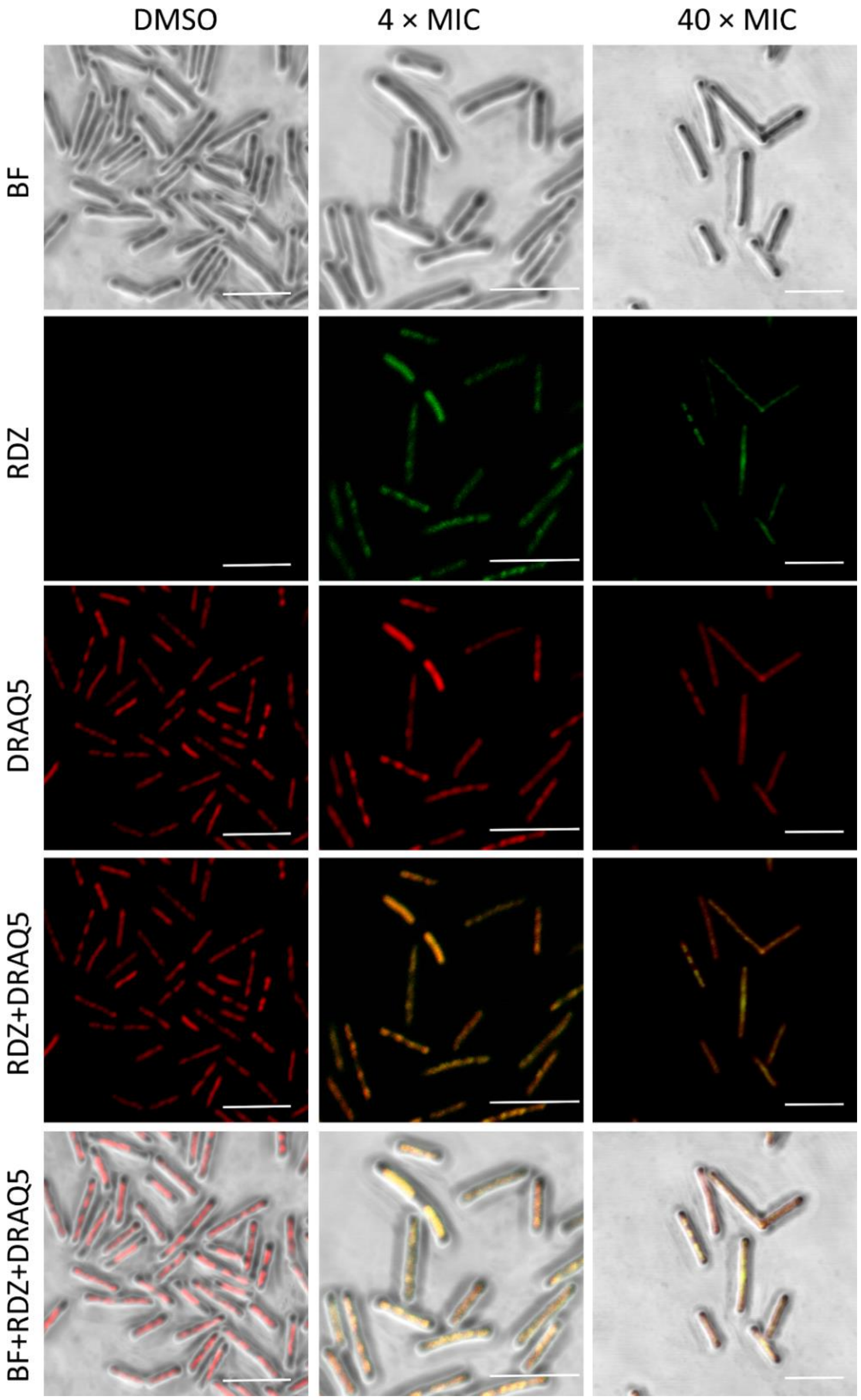

Supplementary Figure 2: Colocalization analysis of ridilinazole (RDZ) and DRAQ5 fluorescence by confocal laser scanning microscopy in the *C. difficile* strain 630. Left, middle, and right panels represent the cells exposed to DMSO, 4 × MIC, 40 × MIC of RDZ for 1 h. All the cells were stained with DNA dye DRAQ5. RDZ fluorescence (shown in green) and DRAQ5 fluorescence (shown in red) were excited by the violet (405nm) and red laser (640 nm), respectively. The fluorescence shown here is not for quantitative analysis purpose since the Lookup Table (LUT) settings were unstandardized among experimental groups. BF denotes the brightfield image whereas "+" denotes the merging mode of two or three different images. Scale bar, 5 µm.

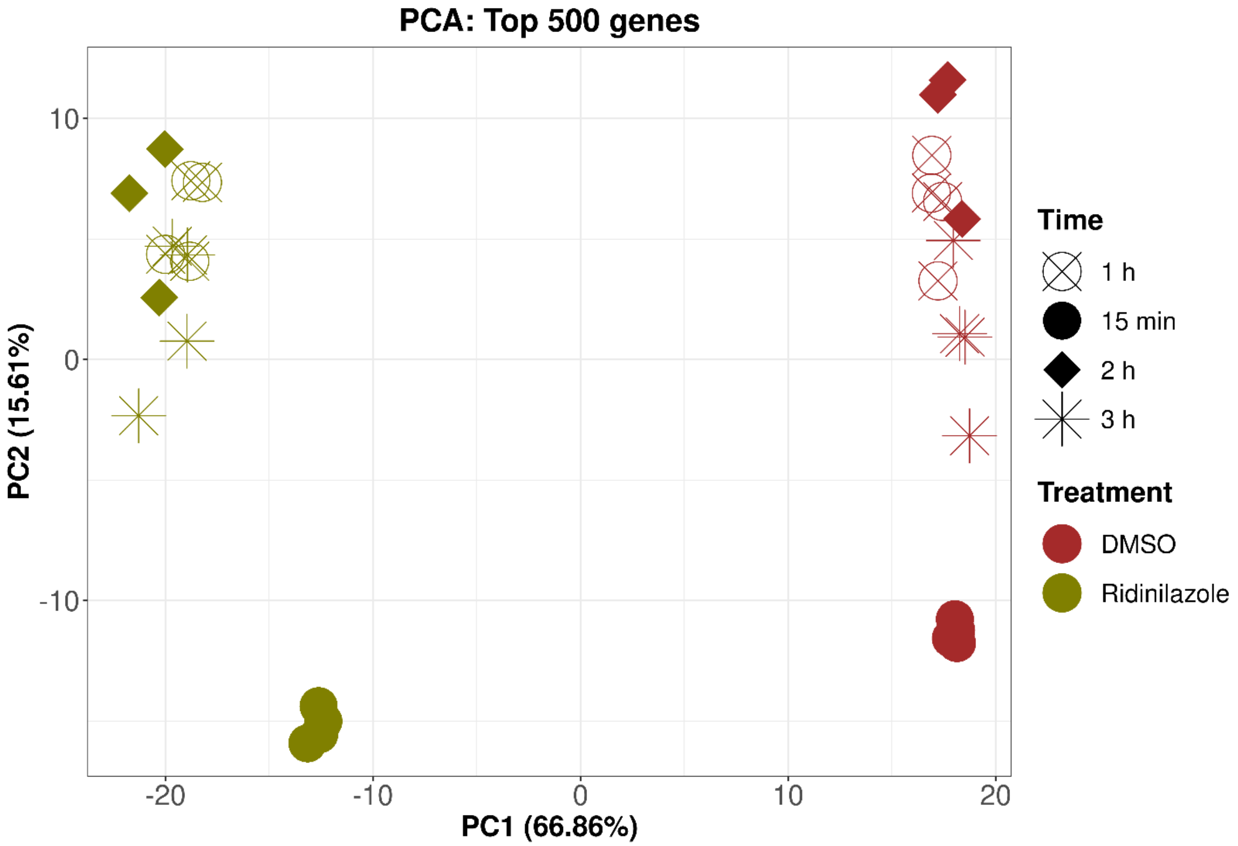

Supplementary Figure 3: Scatter plot of Principal Component Analysis scores from top 500 genes in *C difficile* strain 630 treated with 4x MIC ridinilazole (0.25 µg/mL) or DMSO at 15 min, 1 h, 2 h and 3 h post-exposure.

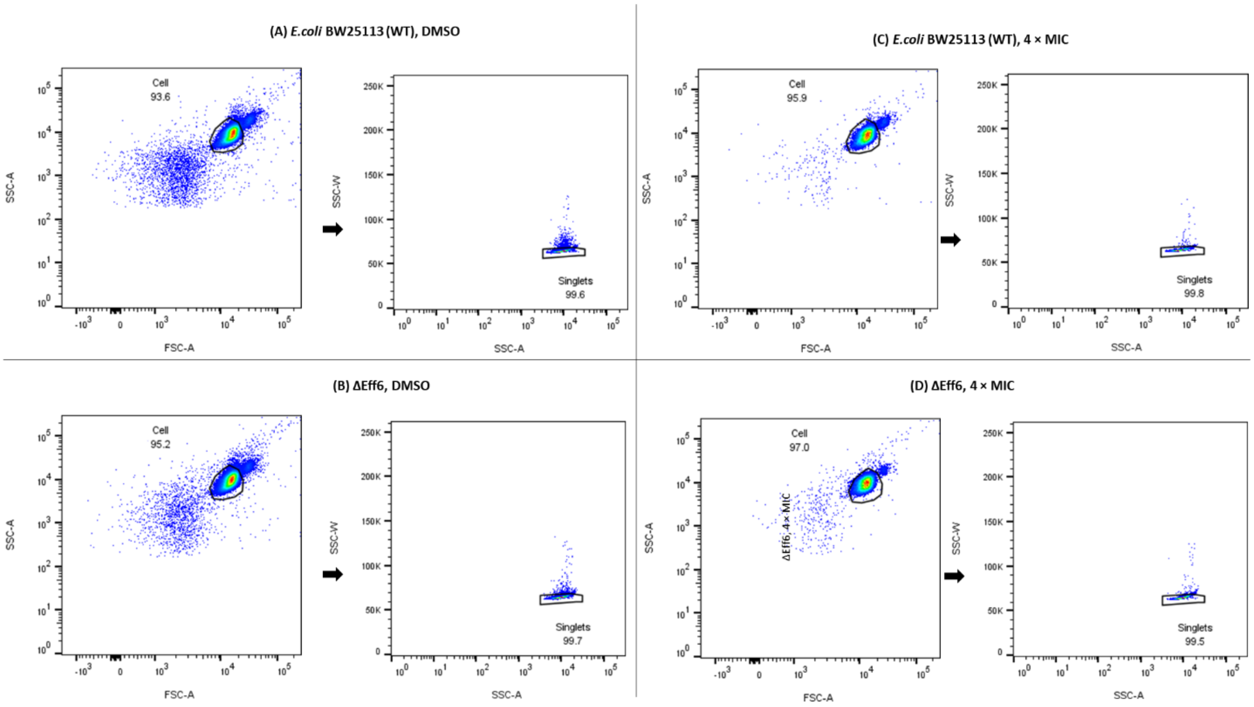

Supplementary Figure 4: Gating strategy for the single-cell population from (A) *E. coli* BW25113 WT and (B) ΔEff6 that were exposed to DMSO for 2h, respectively; (C) *E. coli* BW25113 WT and (D) ΔEff6 that were exposed to 4 × MIC of RDZ for 2h, respectively. The gating strategy was consistent across all four experimental groups. Briefly, to remove the debris, doublets and aggregates, the entire collected objects were firstly gated based on forward scatter area (FSC-A) and side scatter area (SSC-A), and further gated based on side scatter area (SSC-A) and side scatter width (SSC-W). The single cell population (labeled as "Singlets" in figure) was used to plot the histogram of RDZ fluorescence that was excited by the UV laser (355 nm) and detected through the corresponding filter (450/50 BP).

RefSeq Gene ID	Old Locus Tag	Gene Name	UniProt Protein Name	log2FoldChange	padj
CD630_RS12660	CD630_23510	grdB	Glycine reductase complex component B gamma subunit	9.18	2.23E-298
CD630_RS12665	CD630_23520	grdA	Glycine reductase complex selenoprotein A	8.94	0
CD630_RS12655	CD630_23490	grdC	Glycine reductase complex component C subunit beta	8.32	0
CD630_RS12650	CD630_23480	grdD	Glycine reductase complex component C subunit alpha	7.98	0
CD630_RS12685	CD630_23570	grdX	Putative glycine reductase complex component	6.63	0
CD630_RS15845	CD630_29660	adhE1	Aldehyde-alcohol dehydrogenase	3.65	6.91E-135
CD630_RS02220	CD630_03340	adhE	Aldehyde-alcohol dehydrogenase	3.16	2.10E-49
CD630_RS09830	CD630_18060	scrK	Fructokinase	0.55	3.76E-04
CD630_RS10395	CD630_19170	eutE	Ethanolamine acetaldehyde oxydoreductase	0.39	2.35E-01
CD630_RS02520	CD630_03940	ldhA	D-lactate dehydrogenase	-0.24	2.31E-01
CD630_RS01095	CD630_01160	CD630_01160	Putative ferredoxin/flavodoxin oxidoreductase,alpha subunit	-2.10	8.56E-25
CD630_RS01075	CD630_01120	ptb	Phosphate butyryltransferase	-3.06	9.18E-203
CD630_RS17250	CD630_32410	prdB	Proline reductase	-4.62	4.40E-121
CD630_RS17230	CD630_32370	prdF	Proline racemase	-4.64	2.36E-113
CD630_RS17260	CD630_32440	prdA	D-proline reductase proprotein prdA	-4.64	1.68E-181
CD630_RS17240	CD630_32390	prdE	Proline reductase PrdE	-4.91	2.56E-199
CD630_RS17245	CD630_32400	prdD	Proline reductase PrdD	-4.99	1.98E-121
CD630_RS17265	CD630_32450	prdR	Transcriptional regulator, sigma-54-dependent	-0.39	3.74E-03

Supplementary Table 1: List of differentially expressed genes, the fold change, and adjusted p-values between control (DMSO) and ridinilazole-treated (15min) samples associated with the stickland energy metabolism pathway

RefSeq Gene ID	Old Locus Tag	Gene Name	UniProt Protein Name	log2FoldChange	padj
CD630_RS18115	CD630_34050	CD630_34050	Putative iron-only hydrogenase,electron-transferring subunit HymA-like	1.43	1.93E-39
CD630_RS18120	CD630_34060	CD630_34060	Putative iron-only hydrogenase,electron-transferring subunit HymB-like	1.35	1.14E-13
CD630_RS18125	CD630_34070	CD630_34070	Putative iron-only hydrogenase, catalytic subunit HymC-like	1.21	1.55E-15
CD630_RS15820	CD630_29610	CD630_29610	Uncharacterized protein	-2.07	5.23E-17
CD630_RS18450	CD630_34680	atpD	ATP synthase subunit beta	-2.53	1.18E-49
CD630_RS15815	CD630_29600	atpI	V-type ATP synthase subunit I	-2.58	3.86E-67
CD630_RS15785	CD630_29550	atpB	V-type ATP synthase beta chain	-2.65	6.77E-26
CD630_RS15790	CD630_29560	atpA	V-type ATP synthase alpha chain	-2.77	1.95E-47
CD630_RS15780	CD630_29540	atpD	V-type ATP synthase subunit D	-2.85	6.21E-44
CD630_RS15810	CD630_29590	atpK	V-type ATP synthase subunit K	-2.92	4.60E-26
CD630_RS15800	CD630_29570	atpC	V-type ATP synthase subunit C	-3.10	1.61E-38
CD630_RS15795	CD630_29561	atpF	V-type ATP synthase subunit F	-3.14	3.15E-34
CD630_RS15805	CD630_29580	atpE	V-type ATP synthase subunit E	-3.27	5.60E-20
CD630_RS18445	CD630_34670	atpC1	ATP hydrolase epsilon chain	-3.43	7.44E-75
CD630_RS02215	CD630_03330	ppaC	Pyrophosphate phospho-hydrolase	-3.82	6.84E-134
CD630_RS18455	CD630_34690	atpG	ATP synthase gamma chain	-3.86	1.26E-222
CD630_RS18460	CD630_34700	atpA	ATP synthase subunit alpha	-3.94	8.74E-232
CD630_RS18475	CD630_34730	atpE	ATP synthase subunit c	-4.04	1.53E-71
CD630_RS18465	CD630_34710	atpH	ATP synthase subunit delta	-4.34	2.34E-240
CD630_RS18470	CD630_34720	atpF	ATP synthase subunit b	-4.44	5.65E-124
CD630_RS18480	CD630_34740	atpA2 atpB	ATP synthase subunit a	-4.51	9.84E-223

Supplementary Table 2: List of differentially expressed genes, the fold change, and adjusted p-values between control (DMSO) and ridinilazole-treated (15min) samples associated with the ATP synthase energy metabolism pathway

KEGG Pathway ID	Pathway Name	Number of genes in the pathway	Number of Differentially Expressed Genes	p-value
cdf00190	Oxidative phosphorylation	21	18	6.10532E-12
cdf00071	Fatty acid degradation	7	6	0.000124118
cdf02030	Bacterial chemotaxis	19	9	0.00174209
cdf00650	Butanoate metabolism	32	12	0.0035917
cdf00350	Tyrosine metabolism	8	5	0.004503467
cdf00330	Arginine and proline metabolism	19	8	0.007598493
cdf01503	Cationic antimicrobial peptide (CAMP) resistance	9	5	0.008757747
cdf00270	Cysteine and methionine metabolism	36	12	0.010526339
cdf00362	Benzoate degradation	4	3	0.016019523
cdf00625	Chloroalkane and chloroalkene degradation	4	3	0.016019523
cdf01220	Degradation of aromatic compounds	2	2	0.027586965
cdf00626	Naphthalene degradation	2	2	0.027586965
cdf00360	Phenylalanine metabolism	8	4	0.030213499
cdf00280	Valine, leucine and isoleucine degradation	8	4	0.030213499
cdf00260	Glycine, serine and threonine metabolism	20	7	0.036217715

Supplementary Table 3: List of the 15 pathways, from KEGG pathway analysis, implicated in the transcriptional response to ridinilazole exposure (15 min). Table includes the number of genes within a pathway impacted and the associated significance (p-value).

RefSeq Gene ID	Old Locus Tag	Gene Names	UniProt Protein Name	log2FoldChange	padj
CD630_RS03280	CD630_05420	cheB	CheB-type methylesterase domain-containing protein	2.71685482	8.69311E-49
CD630_RS03260	CD630_05380	CD630_05380	Putative methyl-accepting chemotaxis receptor,MCP family	2.662080234	8.4769E-132
CD630_RS03245	CD630_05350	cheD	Probable chemoreceptor glutamine deamidase CheD (EC 3.5.1.44)	2.598560507	7.2997E-167
CD630_RS03265	CD630_05390	cheA	Chemotaxis protein CheA (EC 2.7.13.3)	2.52249066	2.02812E-54
CD630_RS03235	CD630_05330	cheY	Stage 0 sporulation protein A homolog	2.49285952	1.19546E-09
CD630_RS03275	CD630_05410	cheR	Protein-glutamate O-methyltransferase (EC 2.1.1.80)	2.416950923	1.0563E-32
CD630_RS03250	CD630_05360	cheW	Purine-binding chemotaxis protein CheW	2.387450115	1.55075E-42
CD630_RS03240	CD630_05340	cheC	Chemotaxis protein CheY-P phosphatase CheC (EC 3.-.-.-)	2.316945529	2.90123E-16
CD630_RS03270	CD630_05400	cheW	Chemotaxis protein CheW	2.109078421	2.80892E-12
CD630_RS01665	CD630_02280	fliN	Flagellar motor switch protein FliN	1.573903512	0.018097767
CD630_RS02040	CD630_03000	rbsB	ABC-type transport system, ribose-specific extracellular solute-binding protein	1.277013468	2.51066E-06
CD630_RS01885	CD630_02710	fliN1	Flagellar motor switch phosphatase FliN1 (EC 3.-.-.-)	0.466504524	0.00102889
CD630_RS01880	CD630_02700	fliM	Flagellar motor switch protein FliM	0.374123426	0.000260905
CD630_RS01810	CD630_02560	motA	Flagellar motor rotation protein MotA	0.310995095	0.010905751
CD630_RS01770	CD630_02490	fliG	Flagellar motor switch protein FliG	0.306017049	0.018498902
CD630_RS01815	CD630_02570	motB	Flagellar motor rotation protein MotB (Chemotaxis protein MotB)	0.234481983	0.428765416
CD630_RS08675	CD630_15890	CD630_15890	ABC-type transport system, sugar-family extracellular solute-binding protein	-0.0476547	0.94657619
CD630_RS04310	CD630_07440	CD630_07440	Putative MotA/TolQ/ExbB proton channel	-1.12170598	2.72863E-13
CD630_RS04315	CD630_07450	CD630_07450	Putative OmpA/MotB proton channel	-1.123502931	6.92364E-14

Supplementary Table 4: List of differentially expressed genes, the fold change, and adjusted p-values between control (DMSO) and ridinilazole-treated (15min) samples involved in the chemotaxis regulatory network.

RefSeq Gene ID	Old Locus Tag	Gene Name	UniProt Protein Name	log2FoldChange	padj
CD630_RS10315	CD630_18980	CD630_18980	Putative phage-related cell wall hydrolase	3.111973209	1.1682E-93
CD630_RS14885	CD630_27840	cwp6	Putative N-acetylmuramoyl-L-alanine amidase,autolysin cwp6	0.510907912	7.0633E-10
CD630_RS14770	CD630_27610	CD630_27610	Putative N-acetylmuramoyl-L-alanine amidase	-0.511655451	2.0586E-06
CD630_RS04510	CD630_07840	CD630_07840	Putative N-acetylmuramoyl-L-alanine amidase	-1.251027263	2.1413E-10
CD630_RS00820	CD630_01060	cwlD	Germination-specific N-acetylmuramoyl-L-alanine amidase, Autolysin	-1.600695464	1.3972E-14
CD630_RS15225	CD630_28510	dltC	D-alanyl carrier protein	-3.627786035	6.6254E-21
CD630_RS15230	CD630_28520	dltB	Teichoic acid D-alanyltransferase	-4.373081752	2.029E-298
CD630_RS15235	CD630_28530	dltA	D-alanine--D-alanyl carrier protein ligase	-4.468976328	0
CD630_RS15240	CD630_28540	dltD	Protein DltD	-4.577173064	0

Supplementary Table 5: List of differentially expresses genes, the fold change, and adjusted p-values between control (DMSO) and ridinilazole-treated (15min) samples associated with the cationic antimicrobial peptide (CAMP) resistance pathway

	Ridinilazole:dsDNA
**Data collection**	
Space group	P1
Cell dimensions	
a, *b*, *c* (Å)	38.58, 40.32, 40.58
α, β, γ (°)	60.78, 87.31, 61.62
Resolution (Å)	30.08-2.20(2.27-2.20)*
R_{merge}	0.079(0.555)
$I / \sigma I$	5.3(1.1)
Completeness (%)	96.4(87.6)
Redundancy	1.7(1.5)
**Refinement**	
Resolution (Å)	30.10-2.20
No. reflections	8924
R_{work} / R_{free}	0.224 / 0.241
No. atoms	
Protein	0
Ligand/ion	90
Water	48
B-factors	
Protein	NA
Ligand/ion	39.0
Water	39.0
R.m.s. deviations	
Bond lengths (Å)	0.008
Bond angles (°)	2.266

*Values in parentheses are for highest-resolution shell.

Data collection and refinement statistics (molecular replacement)
